# Diabetes: Oral Health Related Quality of Life and Oral Alterations

**DOI:** 10.1155/2019/5907195

**Published:** 2019-03-18

**Authors:** Gabriele Cervino, Antonella Terranova, Francesco Briguglio, Rosa De Stefano, Fausto Famà, Cesare D'Amico, Giulia Amoroso, Stefania Marino, Francesca Gorassini, Roberta Mastroieni, Cristina Scoglio, Francesco Catalano, Floriana Lauritano, Marco Matarese, Roberto Lo Giudice, Enrico Nastro Siniscalchi, Luca Fiorillo

**Affiliations:** ^1^Department of Biomedical and Dental Sciences, Morphological and Functional Images, School of Dentistry, University of Messina, ME, Italy; ^2^Department of Biomedical and Dental Sciences, Morphological and Functional Images, University of Messina, ME, Italy; ^3^Department of Human Pathology in Adulthood and Childhood “G. Barresi”, University of Messina, ME, Italy

## Abstract

**Background and Objectives:**

About 5% of the world's population is affected by diabetes; these patients must be further treated during medical and surgical treatments. These patients, due to the glycemic conditions, realize during their life multiorgan changes, in different body districts. Moreover, this condition obliges them to undertake hypoglycemic therapies. Diabetes is a risk factor for many diseases, including those concerning the oral district with immunological implications.

**Materials and Methods:**

A comprehensive review of the literature was conducted according to PRISMA guidelines accessing the NCBI PubMed database. Authors conducted the search of articles in English language. The results of the last 10 years have been considered, which present useful information regarding the oral conditions. A total of 17 relevant studies were included in the review. The study evaluated only papers with specific inclusion criteria regarding oral health. The works initially taken into consideration were 782; subsequently applying the inclusion and exclusion criteria, there were 42 works. After a careful analysis of the work obtained by two academics who have worked separately, there have been 17 studies. All data from the studies were compared and many of these confirmed alteration in the oral district.

**Results:**

The studies taken into consideration evaluated different factors, such as OHRQoL, QoL, and oral alterations, involving soft tissue, dental structures, and postrehabilitative complications, as well as immunological alterations.

**Conclusions:**

We can affirm, in conclusion, that this study has brought to light those that are complications due to diabetic pathology, from different points of view. The psychological and psychosocial alterations, certainly present in these patients, are probably due to local and systemic alterations; this is confirmed by the correlation between oral health and quality of life reported by the patients.

## 1. Introduction

Diabetes is a term that identifies some diseases characterized by polyuria (abundant production of urine), polydipsia (abundant ingestion of water), and polyphagia (excessive hunger). Commonly the term is used to indicate a chronic disease, which can be included in the group of diseases known as diabetes mellitus, characterized by a high concentration of glucose in the blood, which is in turn caused by a total or partial lack of insulin in the human organism, a hormone that decreases the concentration of glucose in the blood. Diabetes mellitus is a form of diabetes or a group of metabolic disorders united by the fact of persistent instability of the blood glucose level, going from conditions of hyperglycemia, more frequent, at hypoglycemia conditions. Although the term diabetes refers in the common practice to the only condition of diabetes mellitus; that is sweet, there is another pathological condition called diabetes insipidus. The percentage of affected world population is estimated at around 5%. About 90% of the diabetic population is affected by type 2 DM. WHO estimates that there will be a tremendous increase in the prevalence of DM in the USA, the Middle East, and Southeast Asia, while the increase in Europe will be more modest. In 2030, more than 360 million sick people are expected. There was a higher prevalence in women: (m: f = 1: 1.25). A study of the 15-29 year olds with type 1 diabetes had a higher incidence in males than females, perhaps due to factors such as sex hormones or a different exposure to environmental toxins. Complications of diabetes can be different and spread throughout the body. Among the complications of diabetes mellitus we recognize diabetic macroangiopathies, with atherosclerosis phenomena; diabetic ulcers, carpal tunnel syndrome, glaucoma, diabetic neuropathies, cataracts, oral or dermatological infections, and parodontopathies. We also recognize diabetic microangiopathies, therefore nephropathies, retinopathies, and neuropathies of the peripheral nervous system. This increased susceptibility to develop infections in different districts, including the oral one, is of enormous dental interest. In addition there is a predisposition to the development of periodontopathy; all this makes the patient diabetic, a patient to be treated from a dental point of view [1–3].

## 2. Material and Methods

### 2.1. Protocol and Registration

This review was registered on PROSPERO with 120208 ID protocol. PROSPERO includes protocol details for systematic reviews relevant to health and social care, welfare, public health, education, crime, justice, and international development, where there is a health related outcome. Systematic review protocols on PROSPERO can include any type of any study design. Reviews of reviews and reviews of methodological issues that contain at least one outcome of direct patient or clinical relevance are also accepted.

### 2.2. Focus Question

The following focus question was developed according to the population, intervention, comparison, and outcome (PICO) study design:

What are the oral changes present in diabetic patients?

How much does diabetes affect the patient's oral immunological profile?

### 2.3. Information Sources

The search strategy incorporated examinations of electronic databases, supplemented by hand searches. A search of four electronic databases, including Ovid MEDLINE, PubMed, EMBASE, and Dentistry and Oral Sciences Source, Human syndrome for relevant studies published in the English language to 2018 was carried out.

A hand search was also performed in other medical journals. The search was limited to English language articles. A hand search of the reference lists in the articles retrieved was carried out to source additional relevant publications and to improve the sensitivity of the search.

### 2.4. Search

The following key words were used: “Diabetes” AND “Dental" OR “Oral alteration”- “Diabetes” AND “OHIP" AND “Quality of life”- “Diabetes” AND “Dental" OR “Oral” AND “immunologic”. The choice of keywords was intended to collect and to record as much relevant data as possible without relying on electronic means alone to refine the search results. The research was also limited to medical journal and only to articles written in English.

### 2.5. Selection of Studies

Three independent reviewers, of University of Messina singularly analyzed the obtaining papers in order to select inclusion and exclusion criteria as follows. Reviewers compared decisions and resolved differences through comparing the manuscripts. For the stage of reviewing of full-text articles, a complete independent dual revision was performed. The results have been compared at the end of the research. A possible disagreement regarding the inclusion of the studies was discussed among the authors. The first phase of the research consisted of the selection of titles, which allowed us to make a first screening of the manuscript eliminating those not concerning our research. Finally, the full text of all studies was obtained and according to the expected inclusion/exclusion criteria, articles were selected and included in the present review. We obtained a total of 782 results without filters and 256 after the first electronic and manual search with keyword used. We included only 17 full-text English articles on humans.

### 2.6. Types of Selected Manuscripts

The review included studies on humans published in the English language. Letters and editorials were excluded.

### 2.7. Types of Studies

The review included all human use studies and literature reviews published on diabetic patients with focus on oral anomalies, Oral Health Impact Profile, and oral immunological profile.

### 2.8. Inclusion and Exclusion Criteria

The full text of all studies of possible relevance was obtained for assessment against the following inclusion criteria:

- Study of patients with diabetic patients.
- Study of patients with dental anomalies and diabetes
- Study of OHIP or quality of life and diabetes
- Study of oral immunological information and diabetes

The applied exclusion criteria for studies were as follows:

- Studies involving patients with other specific diseases, immunological disorders, oncological patients, osteoporosis, and genetic diseases
- Not enough information regarding the selected topic, no information about oral status and oral health
- No access to the title and abstract in English language or letters, commentary, PhD thesis and editorials
- Animal studies
- Not full-text articles

### 2.9. Sequential Search Strategy

After the first literature analysis, all article titles were screened to exclude irrelevant publications, case reports, and the non-English language publications. Then, researches were not selected based on data obtained from screening the abstracts. The final stage of screening involved reading the full texts to confirm each study's eligibility, based on the inclusion and exclusion criteria.

### 2.10. Data Extraction

The data were independently extracted from studies in the form of variables, according to the aims and themes of the present review, as listed onwards.

### 2.11. Data Collections

Data were collected from the included articles and arranged in the following fields as seen in Table 1:

“Author (Year)” – Revealed the author and year

“Type of study” - Indicates the type of the study

“Sample” – Number of patients and follow up time

“Parameter Evaluated” – Parameter in the study

“Treatment”- Indicates if any treatment has been performed

“Results” - Info about results

“Statistic”- Statistical analysis

Other information is showed in Table 3.

### 2.12. Risk of Bias Assessment

This type of work brings together all the studies in the literature in the last eighteen years presenting a review of recent data about dentomusculoskeletal anomalies in MFS. The risk of bias is minimal as the work is intended to be a collection of works carried out about these patients and about only dental anomalies, but we can only consider full text and abstract accessible articles in English language. Regardless of the results of the studies taken into consideration, the evaluation was carried out on the field of action of the analyses carried out by the studies.

### 2.13. Diabetes

Diabetes mellitus is a chronic disease characterized by an increase in blood glucose concentration. Responsible for this phenomenon is an absolute or relative defect of insulin that allows the body to use glucose for energy processes within cells. When insulin is produced in insufficient quantities from the pancreas or the body's cells do not respond to its presence, glucose levels will be higher than normal in the blood (hyperglycemia) thus favoring the appearance of diabetes mellitus. The diagnosis of diabetes is certain with a blood glucose value of 200 mg/dl, detected at any time of day or two hours after a glucose load. Insulin is a hormone secreted from the islands of Langerhans of the pancreas and essential for the metabolism of sugars. All simple and complex sugars (starches), which are taken with food, are transformed during digestion into glucose, which is the main source of energy for muscles and organs. In order for glucose to enter the cells and be used as "fuel", the presence of insulin is required [3]. The cardiovascular system includes sometimes very serious alterations: the occurrence of an aortic dissection is not uncommon, while a diagnosed patient will be followed throughout the course of the disease and then monitored with an echocardiogram for possible changes in aortic measures. Three subcategories of diabetes mellitus are recognized:

- Type 1 diabetes mellitus (also known as juvenile diabetes):
  - occurs at a young age (within 30 years);
  - the production of insulin is insufficient; therefore, the therapy consists of the administration of insulin;
  - glucose is not used by cells and accumulates in the blood (hyperglycaemia);
  - the high concentration of glucose in the blood prevents the renal tubules from reabsorbing it, with the consequent presence of glucose in the urine;
  - the renal reabsorption of water and sodium is compromised for osmotic reasons dictated by the high quantity of glucose and ketone bodies in the ultrafiltrate, with consequent production of large quantities of urine (polyuria);
  - to the polyuria it follows a strong dehydration that, stimulating the center of the thirst, induces the diabetic to drink continuously (polydipsia);
  - insulin deficiency causes an altered metabolism of fats and proteins, to which is added the inability to store glucose and results in frequent weight loss and increased appetite of the subject (polyphagia);
  - in the long term serious complications occur, especially in the structural and functional alteration of blood vessels (thickening and hardening of the arterial walls, alterations of the capillaries in the retina and kidneys, suffering of the peripheral nervous system).
      Furthermore, type 1 diabetes is further subdivided into the following:
    (1) immune-mediated: it is the diabetic immune system that destroys the beta cells, the only ones responsible for the production of insulin and the regulation of glucose levels. This process of destruction is as rapid as the subject is young (children and adolescents, in fact, can develop ketoacidosis very quickly);
    (2) idiopathic: the individual does not produce insulin and is subject to ketoacidosis, but there are no autoimmune factors involved.
- Type 2 diabetes mellitus:
  - occurs in adulthood;
  - arises due to the inability of cells to use insulin (resistance) correctly and progresses with the gradual loss, by the pancreas, of the ability to produce insulin in adequate quantities;
  - hyperglycemia occurs when the pancreas is no longer able to meet the organic needs and/or when peripheral insulin receptors are compromised;
  - administration of insulin as a therapeutic regimen is often not sufficient and/or indicated, while diet control is fundamental;
  - most people with this type of diabetes are obese;
  - insulin resistance decreases with weight loss, but returns to rise as soon as weight is regained;
  - is subject to strong familiarity.
  - Among the other types of diabetes, we mention the following:
- Gestational diabetes mellitus: type of glucose intolerance that occurs during pregnancy and generally disappears after delivery and returns the patient to normal. It can be managed with or without insulin;
- Secondary diabetes: occurs following other diseases (e.g., genetic defects of beta cells, endocrinopathies, pancreatitis, tumors) or special medical treatments (e.g., corticosteroids);
- Diabetes insipidus: pathology characterized by significant polyuria and insatiable thirst due to an alteration of the production, secretion or functioning of the hormone vasopressin (antidiuretic hormone) at the hypothalamus and pituitary level or from its lack of activity at the renal level.

Among the risk factors for the development of diabetes, a distinction can be made that supports factors on which one can act (obesity, lack of physical exercise) to factors that must be taken into account as appropriate (familiarity with diabetes, age, ethnicity, other pathologies, potentially iatrogenic therapeutic treatments). As we said at the beginning, the acute and chronic complications of diabetes are going to significantly affect the life of the person, since it is a chronic and irreversible disease whose chronicization involves other cascade dysfunctions that are added to the events of

- acute complications: hypoglycemia, hyperglycemia;
- chronic complications: atherosclerosis, retinopathy, nephropathy, neuropathies, diabetic ulcers, increased susceptibility to infections (diabetic foot, phlegmons, cellulitis, necrotizing fasciitis, urinary tract infections, etc.) [1–3].

### 2.14. Hyperglycemia and Immunological Correlation

The diabetic pathology is closely related to immune factors, both as regards its etiopathogenesis and as regards the complications following the overt pathology. According to the most modern and accredited scientific beliefs, insulin dependent diabetes (type 1) is considered a condition by the rather complex and articulated pathogenesis. However, attention is focused on three aspects of the disease, essentially reducible to genetic, environmental, and immune factors. These three aspects, although generally treated separately, are closely intertwined in contributing to the genesis of the disease. In diabetics some alterations of the immune response are present even when the disease is well established over time and, unlike what has been said so far, can appear both in patients with type 1 and type 2 diabetes. In these patients there is a greater susceptibility to infections, most likely due to a deficient immune response [1–9].

One of the most classic explanations refers to the insufficient use of insulin by the cells of the immune system and the consequent decreased activation and differentiation of the same. Therefore, according to this view, diabetes mellitus, when decompensated, would be included among the secondary immunodeficiency conditions. The result is a vicious circle where acute infections in the diabetic promote the metabolic decompensation which in turn reduces the immune response, as periodontitis. Studies in this direction have shown that in many patients with long-term diabetes there is an increase in circulating immunocomplexes, a decrease in the total number of T lymphocytes, and an imbalance in the lymphocyte subpopulations, with a CD4 / CD8 lymphocyte ratio reduced between 1.5 and 2 due in particular to a decrease in cells with CD4 phenotype (helper). Further clinical confirmation of immunological alteration comes from the evidence that patients with disease duration of more than 5 years show a deficient antibody response after vaccination against hepatitis B. The explanation of this phenomenon would be to be found in the nonrecognition of viral antigenic determinants with consequent reduced specific antibody response, due precisely to the alteration of the ratio and/or function of CD4 and CD8 lymphocytes. In the case of anti-influenza vaccination, the antibody titre was instead normal; this could be explained by the fact that as it is a secondary response and because of the cross reactivity with the different viral strains, a clone of T cells with memory already sensitized towards that particular antigen would be stimulated. This process is not strictly dependent, as in the case of hepatitis B vaccination, from a normal CD4/CD8 lymphocyte ratio and therefore clarifies why in the case of influenza vaccination the specific immune response is normal while it is not after the vaccine administration for hepatitis B; in fact in this last case it is a primary answer. In perspective, therefore, we can infer that the normalization of immunological parameters may represent a useful attempt to prevent acute infections, whether viral or bacterial, for the long-term diabetic. In this way it is possible to remove the eventuality of a metabolic decompensation that could derive from it or worse than a resulting ketoacidosis with all the consequences that come from this condition. As is well known, diabetes is a morbid condition that can lead to a series of complications with the increase in the duration of the disease, affecting some organs in a characteristic way. In this regard we mention the diabetic retinopathy which, passing through the phase of simple diabetic retinopathy, can reach the most severe proliferative one, leading in some cases to blindness. The kidney is also a target organ of diabetic disease presenting typical lesions such as the peritubular deposit of glycogen and mucopolysaccharides, arteriosclerosis and glomerulosclerosis. The peripheral nervous system can be affected in practically every district and the lesions may belong either to the type of mononeuropathy, such as poly- or multineuropathy. In addition the autonomic nervous system can also be affected; it is widely accepted that the latter is a fundamental factor in determining and/or accelerating diabetic microangiopathy; however, there are some controversial pathogenetic aspects. In fact, clinical experience teaches us how difficult it is sometimes to find signs of diabetic microangiopathy in patients with poor metabolic control or, on the contrary, there are patients who, despite the optimization of metabolic parameters, present a short distance from the diagnosis of the typical lesions. In this context some immunological aspects are inserted which, although over the years have undergone extensive revisions, maintain a certain validity in the essential lines. Proof of this is the detection of immune complex deposits at the level of the renal capillaries and the increase of the same in the circulation, probably related to the alteration of the mechanisms of "clearance" of the immune complexes. The thickening of the basement membrane, typical lesion in diabetic microangiopathy, would be the consequence, among other things, of a complex series of alterations, among which the entrapment of the anti-andean-antibody complexes would play an important part. From this and many other considerations it emerges that the pathogenesis of diabetic microangiopathy is multifactorial, determined not only on the metabolic side, but influenced by the genetic base and individual variability. Precisely because of this important immunological component, immune therapies have been proposed, to counter both the onset of the disease, but also eventually carry out long-term therapy and limit complications [2, 3, 5–12].

### 2.15. Interdisciplinary Considerations

Periodontal disease (PD) is a chronic inflammatory disease that destroys the gingiva and the tissues surrounding the teeth (periodontal). It is one of the most prevalent chronic infections in adults and affects over 22% of people with diabetes. The two main forms of PD, gingivitis and periodontitis, are the result of bacterial plaque that if destroyed increases the gingival tissue and periodontal. Although it is triggered by bacteria that adhere to the teeth, the individual inflammatory response also plays an important role in the development of periodontal disease: this is why people suffering from chronic diseases such as diabetes, cardiovascular disease, obesity, chronic obstructive pulmonary disease (COPD), and other diseases respirators are much more sensitive and have a greater risk of getting sick. Some studies have documented that periodontitis treatment improves glycemic control, most likely by improving insulin sensitivity. Diabetes and periodontal diseases both have a chronic course but are treatable conditions and should be kept under control with long-term treatment, to ensure better health conditions in general and in the future. In the care of the person with diabetes, the aspect of education and empowerment is of fundamental importance. The diagnosis of diabetes mellitus causes, in fact, the emergence of a situation of fear and insecurity that can negatively affect the correct implementation of the care activities [12–15]. Above all as regards the aspects of containment of anxiety, the intervention of a psychologist is considered necessary both to activate attitudes of coping and to design the training aspects of the patient. Therefore, the themes of psychological interest are identified in the communication phase of the diabetic pathology, in the definition of care interventions and in the phase of support to the patient who is redesigning their existence as a function of this pathology. Diabetes mellitus facilitates the appearance of psychopathological disorders such as depression and anxiety, which in turn affect the management of the disease [13]. The work of the psychologist is an important support in the treatment of the pathology. The practice demonstrated the negative effect that psychosocial dynamics can have on the patient's ability to correctly adhere to therapeutic indications. It is therefore a matter of promoting an attitude of the patient's adherence to therapy and, at the same time, of developing effective coping, making the subject see diabetes as a problem rather than a threat. It follows that good adaptation to the disease depends on the type of individual strategies that the patient puts in place to deal with it. The link between diabetes mellitus and mood disorders is known at least since the 1950s. Symptoms of depression include persistent sadness or inability to experience joy, loss, or increase in appetite, insomnia, apathy, difficulty in concentration, feelings of despair and worthlessness, negative thoughts such as suicidal ideas, irritability, anxiety, nervousness, feelings of guilt. Sometimes depressed people find it difficult to cope with daily programs and activities, and they report significant difficulties in the various fields of life. While depression is very common among the general population, some clinical studies indicate that it is even more common in chronic patients [16–22]. This could be due to numerous factors, including stress resulting from treatment and control of the disease, effects on cognitive function, side effects or complications inherent in drug therapy. There are also both organic and psychological implications in diabetic patients with respect to eating disorders (Table 2) [20–26].

## 3. Results

### 3.1. Study Selection

**Figure 1:**
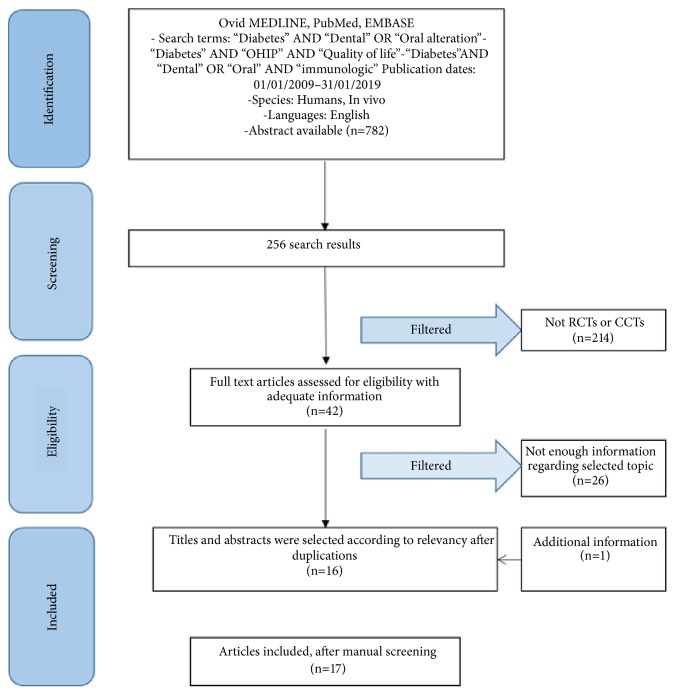
PRISMA flow diagram.

Article review and data extraction were performed according to PRISMA flow diagram (Figure 1). The initial electronic and hand search retrieved 256 citations. 240 papers were excluded because were not identified as full text, RCT, or not enough information about this topic. A study was added later to a manual search, as it contributed to our work. At last only 17 studies were included because of topic reasons.

### 3.2. Study Characteristics

After the study selection a new division related to the kind of bone graft has been performed:

- Diabetes and quality of life
- Diabetes and oral alteration
- Diabetes and immunological alteration

### 3.3. Studies Results

The results were analyzed by the authors independently and were evaluated according to their dental interest field. The review aims to evaluate diabetic patients' symptoms in the medical field, psychology and odontology; all studies have therefore led to satisfactory results. Studies evaluating all types of abnormalities in maxillofacial district are reported. The purpose of this work is to index oral abnormalities and give a rapid diagnostic method proposal for diabetic patients, and for other syndromes early diagnosis.

## 4. Discussion

**Figure 2:**
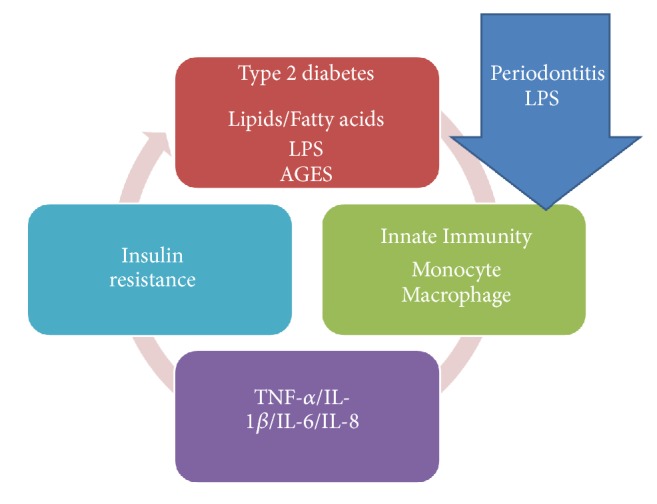
Immunological diabetes aspects and periodontitis.

**Figure 3:**
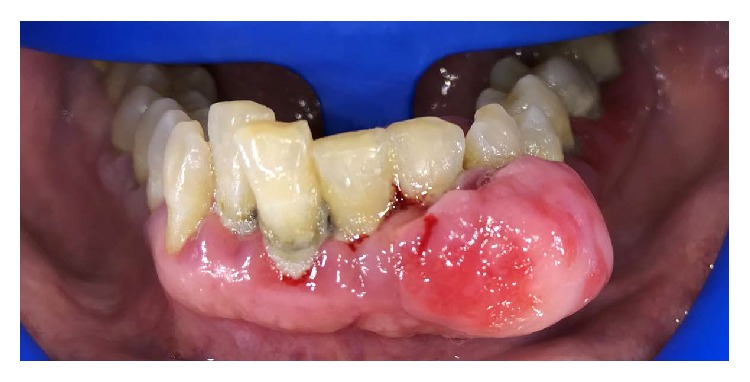
Periodontal disease in diabetic patient, complicated with benign neoformation. This type of lesion, invalidating, also affects the quality of life of the patient, with permission from Dr. L. Fiorillo, 2018.

As widely discussed in the previous chapters, diabetes is therefore a pathology affecting different areas of the organism, also having psychological and social implications. In this article, we have considered the alterations caused by diabetes both at the clinical and immunological levels, while evaluating the alterations on the quality of life of the patients. Cortelli et al. in their study evaluate the impact of gingivitis treatment on oral health and quality of life (OHRQoL). This treatment can improve quality of life and emphasize the relevance of periodontal care for individuals' daily life [27]. Another study shows that treatment intensification, because of diabetes, does not affect adversely patient well-being. Insulin use at entry was associated with longer diabetes duration, worse glycaemic control, and a greater risk of chronic complications; it could influence health status and QoL [28]. Health coaching and health education by dentists, physicians, and diabetes educators in order to improve quality of life is significantly reported [29]. Tzanetakos et al. evaluated the differences, from a cost point of view, about diabetes therapy with insulin Glargine, Liraglutide 1.2mg and exanatide once weekly [30]. Islam et al. evaluated the potential to communicate with diabetes patients and to build awareness about the disease using short message service and monitoring HbA1c and quality of life [31]. Through a diabetes treatment satisfaction questionnaire Vora et al. in their study evaluated differences between glargine/glulisine once daily or insulin aspart/aspart protamine [32]. Evaluation about quality of life and public health costs on diabetic patients with the use of antimicrobial photodynamic therapy (aPDT) and ultrasonic periodontal debridement (UPD) did not show additional benefits for the treatment of chronic periodontitis [33]. Goodson et al. evaluated salivary glucose concentration and other oral factors, like dental caries and gingivitis on diabetic patients. High salivary glucose, in this study was associated with a reduction in overall bacterial load and alterations to bacterial frequencies in saliva on 8173 patients [34]. Another cohort study on 22009 patients, with 3-year follow-up, evaluated periodontitis, dental caries, and peri-implant pathology with exposure to systemic condition prevention. This study reported a high prevalence for oral disease with smoking habits [35]. Oral Health Related Quality of Life, periodontal status, and Oral Health Impact Profile-49 were performed in a Irani et al. study. After nonsurgical periodontal therapy, there was a reduction of gingivitis and periodontitis associated with reduced OHRQoL, with improvements on oral soft tissue health [36]. There is an evaluation about osteonecrosis of the jaw and diabetes during medication. There is a genetic predisposition for MRONJ coupled with CYP 450 gene alteration [37]. Different implant surfaces do not influence implant survival rate on diabetic patients [38]. Cairo et al. evaluated periodontal disease in diabetic patients, showing how diabetes mellitus is an important risk factor for periodontitis [39]; this work was considered, although it did not fall as a year, because it brought an important contribution to our work. There is a correlation between halitosis, HbA1c, and diabetes [40]. The evaluation about reactive oxygen species after antioxidant supplementation evaluated a reduction of ROS levels in patients [41]. Semba et al. in their study showed that high and low AGEs diet did not cause alteration in inflammatory mediators like interleukin-6, C-reactive protein, vascular adhesion molecule-1, and tumor necrosis factor-*α* receptors I and II (Figure 2) [42]. Peripheral blood immune cell subsets after costimulation modulator show that the quantification of CM CD4 T cells can provide a surrogate immune marker for C-peptide decline after diagnosis of diabetes [43]. There are associations between systemic disease and oral alteration like in idiopathic arthritis or other syndromic disease; so these patients need to be attentionated with preventive diagnostic methods (Figure 3) [44–48]. There are no alterations finding about reconstructive technique on these patients [49, 50]. Other studies in literature presented anomalies about peri-implant soft tissue and systemic disease [51]; the approach by the medical staff to these patients is also important, and they can often go against social problems [52].

## 5. Conclusion

In this study, therefore, we analyzed the disease from a general point of view. We evaluated local and systemic complications, and we focused on how this disease affects patients' lives. Surely, there is a lowering of the quality of life reported by patients; this is quite clear from the work we have considered. This lowering of quality of life is often related to local or systemic complications of the disease. We must also remember that there is a close connection between this pathology and the onset of pathologies of psychological interest of the patient. Going to oral alterations, there are clear correlated evidences between diabetic patients and periodontal disease; moreover, this pathology affects the healing of oral surgical wounds in a negative way. Immunological and immunological implications are also evident. There are changes in the proinflammatory cytokines, which still affect healing and the health of oral tissues. In conclusion, we can state that these patients should be further treated, compared to healthy patients, going to evaluate their conditions at 360 degrees in case of oral rehabilitation.

## Figures and Tables

**Table 1:** Most common complications of diabetes [2, 3].

CARDIOVASCULAR DISEASE	SENSORY ORGAN DISEASE	KIDNEY DISEASE	OTHER
(i) Macrovascular disease (ii) Coronary artery disease (iii) Peripheral artery disease (iv) Stroke risks (v) Diabetic foot (vi) Diabetic myonecrosis	(i) Retinopathy (ii) Glaucoma (iii) Cataracts (iv) Blindness	(i) Nephropathy (ii) Chronic kidney disease (iii) Urine protein loss (iv) Tissue scarring	(i) Erectile dysfunction (ii) Periodontal disease (iii) Respiratory infections (iv) Restrictive lung disease (v) Lipohypertrophy

**Table 2:** Psychological and neurological issues related to diabetes [1–3].

NEURO DISEASE	PSYCHOLOGICAL ISSUE
(i) Neuropathy (ii) Muscle atrophy (iii) Cognitive deficit (iv) Diabetic encephalopathy	(i) Cognitive decline (ii) Risk of dementia (iii) Alzheimer (iv) Depression

**Table 3:** Studies evaluated.

Author (year)	Type of study	Sample	Parameter evaluated	Treatment	Results	Statistic
Cortelli et al. (2017) [27]	RCT, double blinded	206 for 3 months	Oral Health and Quality of Life (OHQoL), pocket depth, plaque and gingival indices, PCR for bacteria evaluation, Periotron®	Gingival treatment	OHRQoL improved over time, confirming that quality of life could be changed by the treatment of oral diseases such as gingivitis	P<0.05

Davis et al. (2018) [28]	Observational cohort study	930 (0, 2, 4, 6 years)	Short Form-12 version (SF-12v2), Audit of Diabetes Dependent QoL 19 (ADDQoL)	Blood glucose lowering therapy	These real-life data show that treatment intensification, including insulin initiation, does not impact adversely on patient well-being in community-based type 2 diabetes	P>0.16

Cinar et al. (2013) [29]	Prospective	186	Community Periodontal Need Index (CPI) HbA1c (glycated hemoglobin percentage)	Health coaching (HC), Health Education (HE)	The present findings imply that HC has a significantly higher impact on better management of diabetes and oral health when compared to formal HE	P<0.05

Tzanetakos et al. (2018) [30]	RCT		quality-adjusted life-years (QALYs)	Insulin Glargine vs. Liraglutide 1.2mg vs exanatide once weekly	ExQW was estimated to be cost effective relative to IG or Lira1.2mg for the treatment of T2DM in adults not adequately controlled on OAD	/

Islam et al. (2014) [31]	RCT	216 for 6 months	HbA1c, quality of life	Short message service (SMS)	Mobile phone SMS services have the potential to communicate with diabetes patients and to build awareness about the disease, improve self-management and avoid complications also in resource-limited setting	/

Vora et al. (2015) [32]	RCT	170+165 for 24 weeks	Diabetes Treatment Satisfaction Questionnaire	Glargine/glulisine once daily or insulin aspart/aspart protamine	In long-standing type 2 diabetes with suboptimal glycaemia despite oral therapies and basal insulin, the basal plus regimen was noninferior to biphasic insulin for biomedical outcomes, with a similar overall hypoglycaemia rate but more nocturnal events	

Castro Dos Santos et al. (2016) [33]	RCT	20 at 30, 90, 180 days	Quality of life, public health costs	Antimicrobial photodynamic therapy (aPDT) and ultrasonic periodontal debridement (UPD)	The adjunct application of aPDT to UPD did not present additional benefits for the treatment of chronic periodontitis in type 2 diabetic patients	P>0.05

Goodson et al. (2017) [34]	RCT	8173	Salivary glucose concentration, obesity, dental caries, gingivitis		High salivary glucose was associated with dental caries and gingivitis in the study population	/

de Araújo Nobre et al. (2017) [35]	Open cohort study	22009 for 3 years	Periodontitis, dental caries, and peri-implant pathology	Exposure to systemic conditions was prevented	The present study describes an epidemiological approach to the distribution and determinants of the three principal chronical oral diseases	12.2% less periodontitis and 4.3% less dented caries

Irani et al. (2015) [36]	RCT	61 + 74, 3 to 6 months	OHRQoL, periodontal status, OHIP-49	Nonsurgical periodontal therapy	T2DM does not impact on overall OHRQoL as measured by OHIP-49	there were significantly higher OHIP-49 scores (indicating poorer OHRQoL) in patients with gingivitis and periodontitis

Peer et al. (2014) [37]	RCT	/	Osteonecrosis of the jaw	Medication	Genetic predisposition for MRONJ, coupled with CYP 450 gene alterations, has been suggested to affect the degradation of medications for DM	/

Fontanari et al. (2014) [38]	review	/	Different implant surfaces on diabetic patients	/	It can be concluded that although the benefits of surface modifications present in individuals with diabetes have biological plausibility, there is little evidence of the benefits of these modifications	No significance

Cairo et al. (2001) [39]	Review	/	Periodontal disease	/	Diabetes mellitus is an important risk factor for periodontitis	/

Al-Zahrani et al. (2011) [40]	Review	/	Halitosis status, HbA1c	/	The results of this study suggest an association between halitosis and increased levels of HbA1c	P=0.03

Domanico et al. (2015) [41]	RCT	68	Reactive oxygen species (ROS)	Antioxidant supplementation	Reduction of ROS levels in patients with NPDR thanks to antioxidant therapy	P<0.001

Semba et al. (2014) [42]	RCT	24 for 6 weeks	Peripheral arterial tonometry, serum and urine carboxymethyl-lysine (CML), inflammatory mediators (interleukin-6, C-reactive protein, vascular adhesion molecule-1, and tumor necrosis factor-*α* receptors I and II), soluble receptor for advanced glycation end products (AGEs), and endogenous secretory receptor for AGEs	High or low AGEs diet	A high- or low-AGE diet had no significant impact on peripheral arterial tonometry or any inflammatory mediators after 6 wk of dietary intervention	∖

Orban et al. (2014) [43]		24 months	Peripheral blood immune cell subsets (CD4, CD8-naive, memory and activated subsets, myeloid and plasmacytoid dendritic cells, monocytes, B lymphocytes, CD4(+)CD25(high) regulatory T cells, and invariant NK T cells)	Costimulation modulator	The findings show that the quantification of CM CD4 T cells can provide a surrogate immune marker for C-peptide decline after the diagnosis of type 1 diabetes	/

## Abbreviations

OHRQoL: Oral Health Related Quality of Life
MRONJ: Medication-related osteonecrosis of the jaw
HbA1c: Glycated Haemoglobin
QoL: Quality of Life
ROS: Reactive oxygen species
OHIP: Oral Health Impact Profile.
